# Neuropsychiatric Symptoms in Elderly With Dementia During COVID-19 Pandemic: Definition, Treatment, and Future Directions

**DOI:** 10.3389/fpsyt.2020.579842

**Published:** 2020-09-29

**Authors:** Alessio Simonetti, Cristina Pais, Melissa Jones, Maria Camilla Cipriani, Delfina Janiri, Laura Monti, Francesco Landi, Roberto Bernabei, Rosa Liperoti, Gabriele Sani

**Affiliations:** ^1^ Menninger Department of Psychiatry and Behavioral Sciences, Baylor College of Medicine, Houston, TX, United States; ^2^ Department of Neurology and Psychiatry, Sapienza University of Rome, Rome, Italy; ^3^ Centro Lucio Bini, Rome, Italy; ^4^ Department of Aging, Neurological, Orthopedic and Head and Neck Sciences, Fondazione Policlinico Universitario “Agostino Gemelli” IRCCS, Rome, Italy; ^5^ Service of Clinical Psychology, Fondazione Policlinico Universitario Agostino Gemelli IRCCS, Rome, Italy; ^6^ Department of Geriatric and Orthopedic Sciences, Catholic University of Sacred Heart, Rome, Italy; ^7^ Department of Neuroscience, Section of Psychiatry, Catholic University of Sacred Heart, Rome, Italy

**Keywords:** dementia, COVID-19, apathy, anxiety, agitation, treatment

## Abstract

**Background:**

*Neuropsychiatric* symptoms *(NPS)* of dementia, such as anxiety, depression, agitation, and apathy, are complex, stressful, and costly aspects of care, and are associated to poor health outcomes and caregiver burden. A steep worsening of such symptoms has been reported during Coronavirus Disease 2019 (COVID-19) pandemic. However, their causes, their impact on everyday life, and treatment strategies have not been systematically assessed. Therefore, the aim of this review is to provide a detailed description of behavioral and *psychopathological* alterations in subjects with dementia during COVID-19 pandemic and the associated management challenges.

**Methods:**

A PubMed search was performed focusing on studies reporting alterations in behavior and mood and treatment strategies for elderly patients with dementia, in accordance with *PRISMA* guidelines. The following search strategy was utilized: (COVID* OR coronavirus OR “corona vir*” OR SARS-CoV-2) AND (dementia OR demented OR dement* OR alzheimer* OR “pick’s disease” OR “lewy body” OR “mild cognitive” OR mild cognitive impairment OR MCI).

**Results:**

Apathy, anxiety and agitation are the most frequently *NPS* during the COVID-19 pandemic and are mainly triggered by protracted isolation. Most treatment strategies rely on pharmacotherapy; technology is increasingly utilized with mixed results.

**Conclusions:**

*NPS* of dementia during COVID-19 appear to arise from social restrictions occurring as a consequence of the pandemic. Implementation of caregiver support and the presence of skilled nursing home staff are required to restore social interaction and adjust technological support to the patients’ needs.

## Introduction

In late 2019, a new respiratory syndrome, now known as coronavirus disease 2019 (COVID-19), was reported in Wuhan, China (1). The identified cause was a novel coronavirus, the severe acute respiratory syndrome coronavirus 2 (SARS-CoV-2). Since then, infection from SARS-CoV-2 has spread globally, officially becoming a pandemic on March 11, 2020 (2).

The increasing mortality rates from SARS-CoV-2 stressed global healthcare systems, prompting the vast majority of countries to adopt extraordinary measures to limit contagion spread *via* the enforcement of social distancing, quarantining of people exposed to the disease, and confinement of the healthy at home except for essential outings (3).

The majority (75%) of people affected by COVID-19 recover without treatment (4). However, mortality increases with age (5) and the presence of comorbidities (6). Among them, dementia is associated with greater risk of death (7). Increased risk of death in elderly patients with dementia impairment may not be solely due to their vulnerability to SARS-CoV-2 infection (8), but may also relate to the cognitive, behavioral and psychological effects of rapid environmental changes brought by the pandemic. Worsening of cognitive impairment in elderly patients with dementia has been reported during the few months following the beginning of the pandemic (2, 3, 9). Impaired comprehension of the public health situation and difficulty following restrictive measures has also been reported (10). More importantly, several authors have described a steep worsening of a plethora of neuropsychiatric symptoms (NPS), including depression, anxiety, anger, agitation, insomnia (11). These complications may increase levels of distress in caregivers and nursing home staff (12), favor contagion (2), and increase risk of self-injury, hospitalization, and death (13). Managing NPS in elderly patients with dementia is particularly challenging during the COVID-19 pandemic in the context of lacking routine infection screening programs (2), isolation from family members who would otherwise visit and monitor the status of their loved ones (14), and a general deficiency in the widespread implementation of non-pharmacological treatments for dementia (15).

Given this stress on healthcare systems and caregivers, systematic description of the psychopathology arising during COVID-19 pandemic in elderly patients with cognitive disorders and possible treatment strategies, are greatly needed to guide management. Therefore, the aim of this review is to describe the behavioral and psychopathological characteristics of elderly patients with dementia during the COVID-19 pandemic and potential interventions.

## Methods

A PubMed search was performed of all literature published before June 19, 2020 using the following terms: (COVID* OR coronavirus OR “corona vir*” OR SARS-CoV-2) AND (dementia OR demented OR dement* OR alzheimer* OR “pick’s disease” OR “lewy body” OR “mild cognitive” OR mild cognitive impairment OR MCI). The search was performed by two researchers (GS and CP) independently. Papers included in this review met the following criteria: (i) written in English; (ii) an original article (no review or meta-analyses were allowed); (iii) focused on subjects with dementia of any etiology (e.g. Alzheimer’s Disease (AD), Pick Disease, Lewy body disease); (iv) included geriatric populations; (v) reported original data, case series, or case reports, and (vi) provided information of the characteristics and/or recommendations for the management of *NPS* in subjects meeting the aforementioned criteria during COVID-19 pandemic. Exclusion criteria were: (i) reviews and meta-analyses; (ii) editorials, comments, notes or letters without any data and/or recommendations; (iii) studies with aims inconsistent with the scope of the review (e.g. studies investigating behavioral problems in the elderly without cognitive impairment); (iv) studies focusing on non-elderly populations; (v) studies specifically designed to describe the scope and rationale of a multicenter study (defined as “rationale”); (vi): articles without peer-review or in which peer-review process is still pending (defined as “preprint”); (vii) studies not including human subjects (defined as “*in vitro*”).

Inclusion and exclusion of papers were based on consensus discussion among the two researchers performing the aforementioned research and the among all authors; unanimity was required for both and was achieved through Delphi rounds. Two rounds were sufficient to reach complete agreement for paper inclusion or exclusion.

This review follows the *P*referred *R*eporting *I*tems for *S*ystematic reviews and *M*eta-*A*nalyses (*PRISMA*) guidelines (16). A *PRISMA* checklist and flowchart as well as detailed results stemming from database searches are shown in the Online Supplement.

## Results

**Figure 1:**
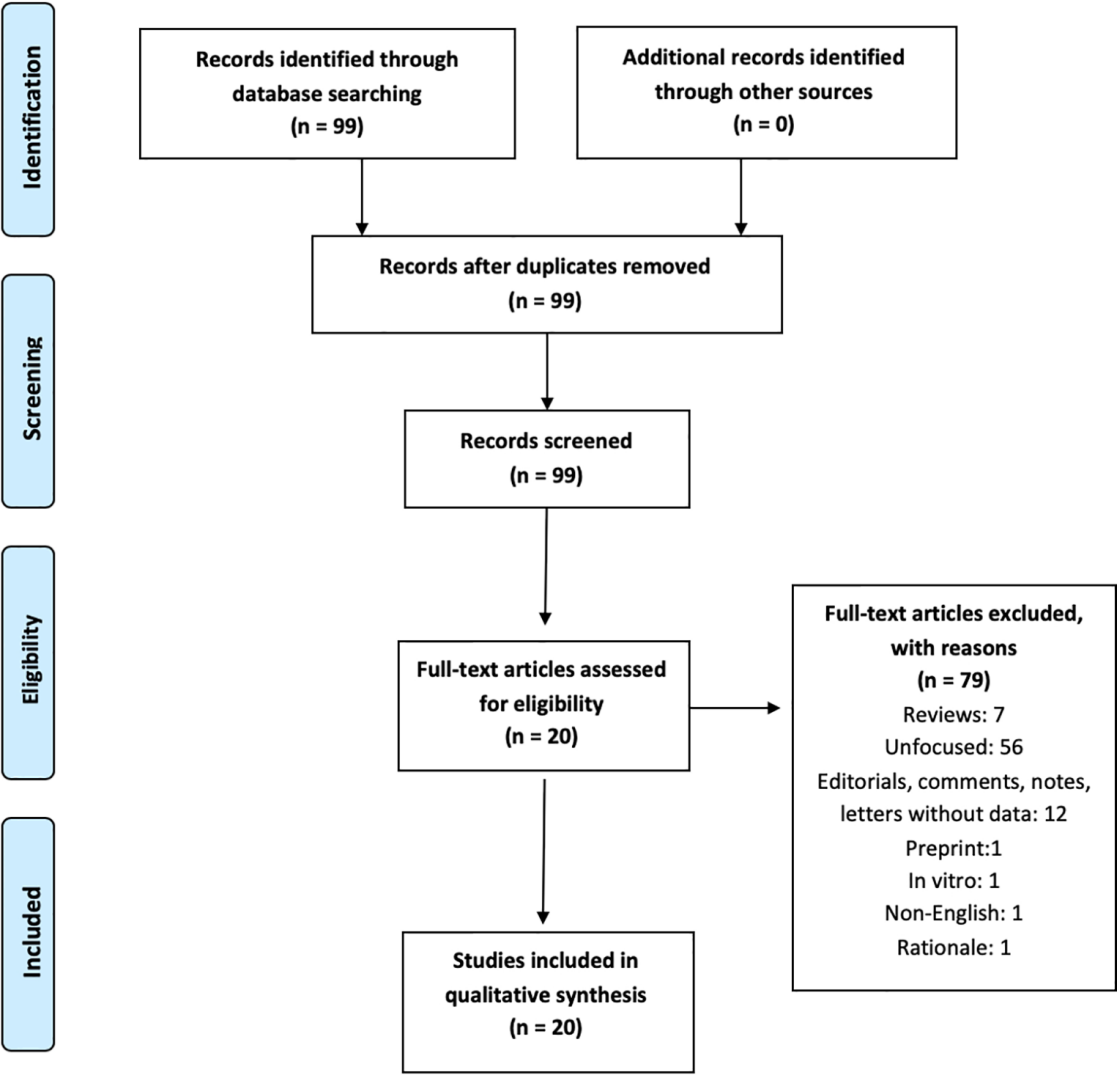
*PRISMA* flowchart of our review’s results.

The search produced 99 records on June 19, 2020. Dates of publication of such 99 records spanned from 1960 to 2020. A total of 20 papers were eligible following application of the inclusion/exclusion criteria and consensus determination. Eligible studies spanned from March 2020 to June 2020. Therefore, these dates represent the period of enrollment of this research. Reason of exclusion are shown in **Figure 1**. Results are described below according to the type of NPS and treatment issues/strategies.

### Symptoms

#### Mood

Evidence of mood alterations in subjects with dementia during COVID-19 pandemic is mixed. Development of depressed mood, hopelessness and increased suicidal ideation is anecdotally described (13), mainly due to protracted isolation and loss of familial contact due to confinement in homes or in nursing facilities (2). Accordingly, Canevelli and colleagues (15) reported an increase of depressed mood within the first month of lockdown in an Italian sample of subjects with dementia. On the other hand, in a similar cohort in Spain, no worsening of depression was observed after 5 weeks of home confinement (9). Additionally, increased hopelessness was reported in subjects with AD after interruption of experimental trials on potential disease-modifying drugs. This behavior was induced by the sudden withdrawal of the social support from clinical care staff and their participation in the trial (17). The onset or worsening of elation/euphoric mood was poorly reported, and when it was, the occurrence of it was low (15).

#### Apathy

Apathy, i.e. a general absence of motivation or interest in activities, appears to be consistently impacted by persistent isolation in subjects with dementia during the COVID-19 pandemic. In a survey of 300 psychologists or healthcare practitioners working in nursing homes, apathy was reported to be the most common behavioral disturbance manifesting from protracted isolation due to COVID-19-related social restriction in subjects with AD (18). Accordingly, Canevelli and colleagues (15) reported that apathy presented in more than 25% of quarantined subjects with dementia within the first month of lockdown in Italy. Apathy also overwhelmingly increased over time, as compared with depressed mood, in subjects with dementia and home confinement (9). Patients with apathy are less likely to initiate behaviors necessary to impede the transmission of the virus, including selfcare and personal hygiene, washing hands, or covering their mouth while coughing (2). Protracted apathy might also lead patients to spend more time in beds, thus increasing the risk of pressure ulcers and hospitalizations (13).

Apathy occurring in the context of altered consciousness was also described as an atypical presentation of the SARS-CoV-2 infection (11). This so-called “apathetic delirium” may supersede classical SARS-CoV-2 infection symptoms and interfere with the early identification of COVID-19 disease (11, 19).

#### Anxiety

Anxiety and aggression were reported as the main psychopathological manifestations in patients with AD in an Alzheimer’s clinic in France during the COVID-19 pandemic (20). During the same period, in a multicenter European study of isolated-at-home subjects with dementia, greater levels of anxiety differentiated those living alone to patients living with at least one family member. This suggests that anxiety is particularly fostered by a reduction in social contact (3). Abrupt withdrawal of social contacts has been also reported to foster anxiety related trauma experiences, which in turn have been found to accelerate cognitive decline and worsen prognosis (2). Anxiety related to isolation also dominated the clinical presentation in a woman affected by early-dementia (21).

#### Motor Activity

Agitation is another typical behavioral alteration described in confined subjects with dementia (15) during the COVID-19 pandemic. Motor agitation also steeply worsened over time in subjects with AD (9) and high levels of motor agitation and fear were reported in a Dutch survey of patients living in nursing facilities during the pandemic (18). High levels of agitation need to require greater dosages of medication to maintain behavioral control (22). Greater motor activity was also associated with intrusiveness or wandering, which may undermine efforts to maintain isolation, thus increasing the risk of contagion (2). Motor retardation is not reported, at least as an isolated symptom, possibly due to its characterization as a manifestation of apathy or depression (23).

#### Appetite

Loss of appetite is frequently reported in relation to isolation. In particular, this symptom appears to coincide with social restrictions during COVID-19 pandemic, especially in nursing homes. In these environments, such behavior may persist even when family members were asked to prepare the patient’s favorite meal (13). The interruption of activities facilitating feeding and social life (e.g., sharing meal-time in nursing facilities or assistance with eating) induced by the pandemic has been proposed as a factor influencing loss of appetite and malnutrition, especially in the COVID-19 era (13). Loss of appetite and malnutrition may also increase risk of hospitalization.

#### Circadian Rhythms

Sleep alterations often accompany agitation in isolated subjects with dementia (22). Reduced quality of sleep is reported in subjects living alone compared to subjects not living alone during COVID-19 confinement (3). Importantly, sleep alterations may be particularly dangerous due to their potential to increase the risk of delirium, and accordingly, the risk of mortality (2).

#### Psychotic Symptoms

Data on psychotic symptoms without alteration of consciousness are infrequently reported during the COVID-19 pandemic. Lara and colleagues (9) reported no changes in hallucination/delusion severity in elderly with dementia after 5 weeks of social isolation. On the other hand, paranoia was associated with rapid changes in social context (i.e. from in-person contacts to video calls) during the quarantine (18).

### Treatment Challenges

#### Pharmacological and Non-Pharmacological Strategies

Some authors provide recommendations aiming to reduce behavioral dyscontrol in subjects with dementia in accordance with recommendations from dementia association guidelines, accounting for limitations caused the pandemic (24). Such guidelines stress implementation of technology to improve mood; maintain daily activities at home (e.g., gardening, cooking, reading, listening to music, physical exercise) to treat apathy; and foster the development of simplified and sequential routines to treat anxiety. However, the shrinkage of support commonly provided by caregivers, nursing home staff, and environmental resources heavily limits the efficacy of these non-pharmacological strategies.

On the other hand, a surge in the dosage of medications commonly needed to treat NPS, such as antipsychotics and mood stabilizers, has been reported (15, 25). For instance, a double dose of loxapine was needed to control behavior of an elderly patient with dementia and severe agitation (26). However, increases in pharmacological treatment strategies during COVID-19 pandemic carries several risks. First, several authors report an inability to increase or change drug dosages due to the disruption of routine assessments, including in-person clinical visits, blood work, or electrocardiograms, or the inability to follow up on adverse events in a timely manner (2).

Moreover, the increased utilization of antipsychotics (specifically without monitoring) may double the risk of death and triple the risk of stroke (27, 28). In order to avoid increasing antipsychotic usage and dosages, physical restraint techniques have been used to control agitation (26). Other specific programs, which included selective, personalized isolation for those who cannot comply with current isolation guidelines, have been described (29, 30). However, behavioral dyscontrol in patients with dementia largely exceeds the resources provided by nursing homes (31), and systematic application of personalized isolation may be difficult to implement.

#### Use of Electronic Devices

A second theme described in the literature is the management of isolation and prevention of its associated behavioral dyscontrol through the use of technology. In many nursing homes, as well as in personal home settings, the use of electronic devices has been increasingly utilized to maintain patients’ social supports and monitor their clinical state by healthcare providers (32). However, the effectiveness of the use of electronic devices in patients with dementia is mixed. Due to the inability of electronic devices to facilitate adequate physical and neurological examinations necessary for diagnosis and follow up, technology platforms may lead to incorrect assessments of cognitive and behavioral statuses in cognitively-impaired elderly (2). Prevalent hearing and vision problems in subjects with dementia may also interfere with interpretation of such assessments (33). Despite some authors advocating for the electronic provision of information on physical exercise, sensory stimulation, reminiscience-based brain health, music therapy, and other creative activities for people with dementia in the home (34), Goodman-Casanova and colleagues (3) found that the implementation of these approaches did not produce behavioral improvements over time. On the other hand, Padala and colleagues (22) reported a case in which depressive symptoms and agitation in a subject with dementia in a nursing home improved after the patient was able to see his family through facetime. In another case report, a woman affected by dementia relieved anxiety by using computer and social media applications (21).

In recognition of the importance of in-person contact and caregiving, the Dutch Alzheimer Association requested permission from the government to allow one visit per patient per day in nursing homes during the early weeks after the lockdown in the Netherlands. While initially denied by the government, visits were subsequently allowed once the number of affected subjects in Netherlands dropped (18).

## Discussion

**Figure 2:**
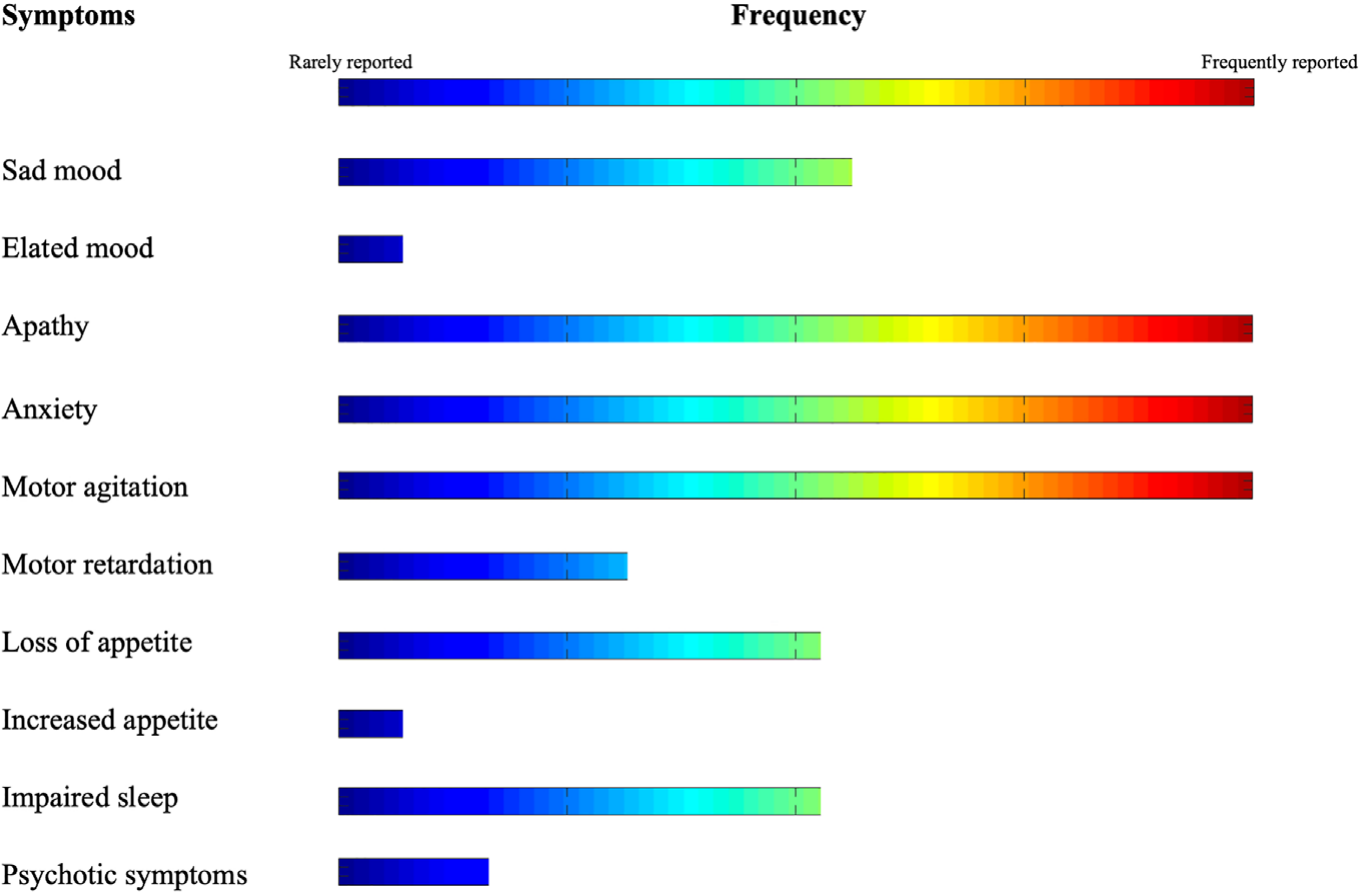
Frequency of *NPS* in elderly with dementia during COVID-19 pandemic.

In this review, we described the findings of recent literature on the nature, trajectory, and management strategies of NPS during COVID-19 pandemic in subjects with dementia. Our search indicates that NPS in the COVID-19 era span from inhibition of volition, movement and initiative (i.e., apathy) to severe hyperactivity (i.e., anxiety and agitation) (see **Figure 2**). On the other hand, treatment strategies frequently rely on pharmacological interventions to control behavior. On the other hand, technology is used as a compensatory strategy to counterbalance the drastic lack of non-pharmacological interventions.

Anxiety, agitation and apathy are cardinal behavioral and psychological features of dementia (35). During COVID-19 pandemic, they appear to worsen after protracted isolation due to environmental restrictions. Isolation may foster behavioral disturbances *via* several, partially overlapping mechanisms. Forced separation may interfere with caregiver support, whether the subject lives in his/her house or in nursing homes (24), and leads to poor health monitoring. Loss of interpersonal monitoring may increase the risk of dehydration, infections, and the decompensation of chronic diseases, such as diabetes or hypertension (36). As patients with dementia may show impairments in the interpretation and outward expression of stimuli in their internal *milieu* (37), symptoms related to possible medical conditions, such as pain, may be expressed *via* aberrant arousal and motor responses, i.e., anxiety and agitation (38, 39). Apathy is also known to be exacerbated by acute medical conditions. Worsening of physical status causes rapid acceleration of impairments in cognitive functions. Such decline has been shown to be paralleled with an increase of apathy (40), possibly through progressive prefrontal based circuitry dysfunction (41–43). However, emotional distress might trigger anxiety, agitation or apathy (44, 45). Similarly, sadness and hopelessness have been reported in isolated elderly with dementia (13). Therefore, a more direct, psychological effect of isolation on behavioral and psychological alterations in dementia cannot be excluded.

The available evidence suggests that the management of NPS during COVID-19 should ideally rely on non-pharmacological interventions (46). Non-pharmacological strategies consist of: a) patient-targeted interventions, including several techniques aimed at reducing stress (47–50); b) caregiver-targeted interventions, which primarily consist of support programs and training to enhance problem solving (51, 52); c) environment-targeted interventions, which include plans aimed to reduce potentially destabilizing aspects of patients’ surroundings, such as environmental over- or under-stimulation, safety risks, or a lack of routine (53–55). Unfortunately, environmental changes induced by the COVID-19 pandemic undermines the foundation of all these interventions. Limitation in gatherings impedes activities aimed to enhance social life, autonomy, and cognitive abilities. Furthermore, social contact restrictions minimize caregiving support from patients’ relatives or nursing home staff (56). In fact, strict behavioral rules brought by the COVID-19 pandemic (respect for hygiene, the use of masks and the maintenance of social distancing) increase the caregiver’s daily workload, with consequential barriers to providing routine support (57). Caregiving, either by family members or nursing home staff, is even more difficult in the context of infection risk. In fact, the social contact required to perform the act of caregiving may n heighten the caregiver’s fear of getting sick, being unable to assist family members, and/or of potentially infecting them. Together, these stressors increase the risk of caregiver distress and anxiety (58). Issues in managing the elderly with dementia are present also in nursing home and they are compounded by the inability to quickly provide infrastructure, technology and the skilled staff required to tend to patients’ needs during isolation (56). These barriers to the implementation of non-pharmachological strategies may result in the use of pharmacological treatments. However, pharmacotherapy may not be effective for anxiety or sad mood in patients with dementia (59). Pharmacotherapies are also associated with several side effects, such as drowsiness, extrapyramidal symptoms, orthostatic hypotension, (60–63), and higher risk of death (64–66).

**Figure 3:**
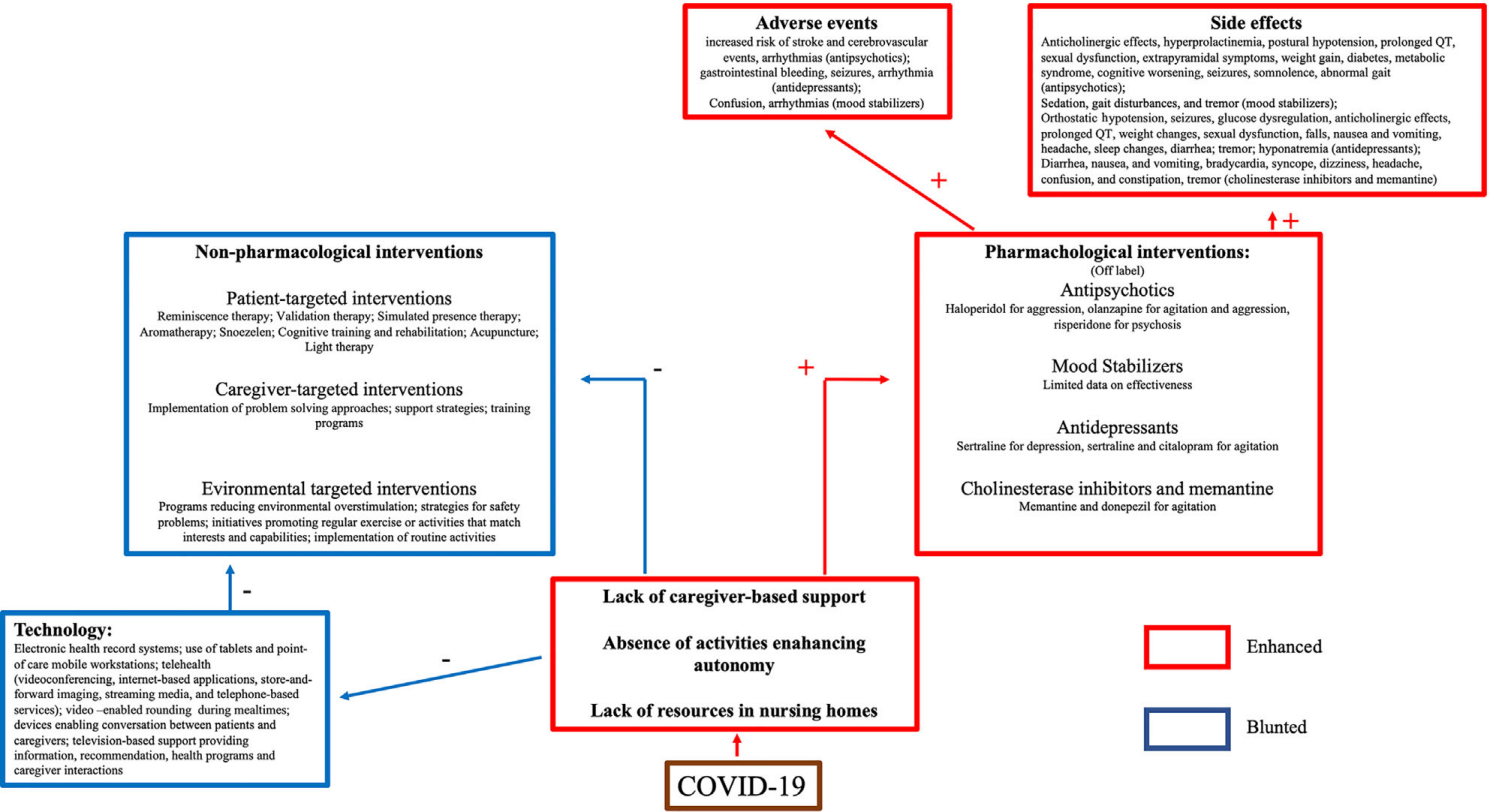
Schematic representation of treatment challenges in patients with dementia during COVID-19 pandemic.

The application of technology may be the most realistic solution to address the need for non-pharmacological supporting the cognitively impaired elderly. However, despite some enthusiastic reports (67), findings are generally mixed. One limitation of technological applications is the inability to train caregivers on the use of computer-based support strategies (68) due to lockdown-related restrictions or a lack of skilled staff in nursing homes. Caregivers are required to address the needs of the user and the user’s acceptance of technology (14). Acceptability, i.e., the degree of primary users’ predisposition to carry out daily activities using the intended device (69), is based on a complex interaction between the subjects’ confidence with the technology, the caregiver beliefs, and the time spent in training (70, 71). Without caregiver support or training, patients may not view the device as useful, or the patient may feel stigmatized (72). These issues may have influenced the results of the studies reporting the use of technology to address behavioral dyscontrol during the pandemic (see **Figure 3**).

Therefore, specialized programs and support are needed to address the escalation of behavioral dyscontrol observed in elderly with dementia during the pandemic. Implementation of environmental and caregiver supports are required to facilitate the use of technology. Additionally, services promoting social interaction should be restored as soon as possible.

## Limitations

The urgency to provide a comprehensive review of NPS occurrence and management during the Covid-19 pandemic, in combination with the scarcity of high-quality of studies, led us to include case reports, case series, recommendations or anecdotal reports. Therefore, conclusions drawn from this review should be interpreted with caution. Specifically, factors proposed to cause a surge/worsening of NPS, i.e. blunting of social activities and insufficient caregiving brought by isolation, should be considered as highly speculative. The rapid spread of COVID-19 and the consequential lack of long-term follow-up studies impede a clear disentanglement of the effects of isolation from other possible, co-occurring influences. As stated above, NPS, and specifically apathy, might represent the most relevant symptom of acute COVID-19 infection (11). Additionally, SARS-CoV-2 infection has been proposed to directly induce neurodegeneration, even though specific studies investigating such mechanisms in the elderly, and specifically in those with a well-defined diagnosis of dementia, are absent (73). Therefore, NPS might surge/worsen due to a direct effect of SARS-CoV-2, rather than being an indirect consequence of COVID-19 pandemic-related isolation. Accordingly, the aforementioned issues prompt us to underline the preliminary nature of the treatment paradigms proposed by this review. The effectiveness of antidepressants, methylphenidate, memantine, low dosages of atypical antipsychotic, as well as non-pharmacological interventions in treating NPS has been extensively investigated in subjects with dementia in the pre COVID-19 pandemic era (74–76), whereas evidence supporting recommended strategies during COVID-19 pandemic are still based on limited data. Additional studies with larger sample sizes, longer follow-up durations, or placebo-controlled designs are needed to clearly define the impact of COVID-19 disease on NPS, the cause of the surge in NPS, and appropriate treatment strategies during this time period. Furthermore, the selected studies were unable to provide comparisons of symptoms among different forms of dementia, such as AD or frontotemporal dementia, or different environments, i.e., between subjects living in their house or in nursing homes. Therefore, discussions are limited to subjects with dementia (considered as a whole), and we cannot provide recommendations for specific sub-populations. Finally, the conclusions drawn in this review are biased by the unclear assessments and definitions of social isolation in the selected studies. Social isolation and social functioning should ideally be assessed by combinations of objective and subjective self-report measurements (77). The development of standardized methodologies of assessing social isolation would provide much needed clarity to the study of the behavioral sequelae of pandemic-related social restrictions.

## Conclusions

The COVID-19 pandemic has disrupted everyday life. This interruption of routine activities is particularly dangerous in the cognitively impaired elderly due to their sensitivity to environmental changes. Disruption of routine may lead to the onset/worsening of *NPS* that increase the risk of self-injury, personal distress, COVID-19 contagion, and death. The use of technology may represent a valid alternative to in-person social contact and facilitate non-pharmacological interventions. However, the use of technology is limited by the requirement for a caregiver to customize the technology to the patient’s needs.

## Data Availability Statement 

The original contributions presented in the study are included in the article/supplementary material; further inquiries can be directed to the corresponding author.

## Author Contributions

GS and AS designed the review, all authors were involved in selection of eligible material and in Delphi rounds to reach consensus. AS and CP wrote the *Introduction*, *Methods*, and *Results*, designed the search strategy, gathered eligible material, and supervised the writing of the paper along with RL, LM, FL, and RB. DJ and MCC wrote the *Discussion*. MJ and LM wrote the *Limitations* and *Conclusions*. All authors contributed to the article and approved the submitted version.

## Conflict of Interest

The authors declare that the research was conducted in the absence of any commercial or financial relationships that could be construed as a potential conflict of interest.

## References

[1] Lu H Stratton CW Tang YW Outbreak of pneumonia of unknown etiology in Wuhan, China: The mystery and the miracle. J Med Virol (2020) 92(4):401–2. 10.1002/jmv.25678 PMC7166628 31950516

[2] Brown EE Kumar S Rajji TK Pollock BG Mulsant BH Anticipating and mitigating the impact of the COVID-19 pandemic on Alzheimer’s disease and related dementias. Am J Geriatr Psychiatry (2020) 28(7):712–21. 10.1016/j.jagp.2020.04.010 PMC7165101 32331845

[3] Goodman-Casanova JM Dura-Perez E Guzman-Parra J Cuesta-Vargas A Mayoral-Cleries F Telehealth home support during COVID-19 confinement for community-dwelling older adults with mild cognitive impairment or mild dementia: survey study. J Med Internet Res (2020) 22(5):e19434. 10.2196/19434 32401215 PMC7247465

[4] Del Rio C Malani PN COVID-19-New insights on a rapidly changing epidemic. JAMA (2020) 323(14):1339–40. 10.1001/jama.2020.3072 32108857

[5] Abbatecola AM Antonelli-Incalzi R Editorial: COVID-19 spiraling of frailty in older italian patients. J Nutr Health Aging (2020) 24(5):453–55. 10.1007/s12603-020-1357-9 PMC7136701 32346677

[6] Wang D Hu B Hu C Zhu F Liu X Zhang J Clinical characteristics of 138 hospitalized patients with 2019 novel coronavirus-infected pneumonia in Wuhan, China. JAMA (2020) 323(11):1061–69. 10.1001/jama.2020.1585 PMC7042881 32031570

[7] Covino M De Matteis G Santoro M Sabia L Simeoni B Candelli M Clinical characteristics and prognostic factors in COVID-19 patients aged ≥80 years. Geriatr Gerontol Int (2020) 20(7):704–8. 10.1111/ggi.13960 PMC7300699 32516861

[8] Garcia-Ptacek S Farahmand B Kåreholt I Religa D Cuadrado ML Eriksdotter M Mortality risk after dementia diagnosis by dementia type and underlying factors: a cohort of 15,209 patients based on the Swedish Dementia Registry. J Alzheimers Dis (2014) 41(2):467–77. 10.3233/JAD-131856 24625796

[9] Lara B Carnes A Dakterzada F Benitez I Piñol-Ripoll G Neuropsychiatric symptoms and quality of life in Spanish patients with Alzheimer’s disease during the COVID-19 lockdown. Eur J Neurol (2020) 27:1744–7. 10.1111/ene.14339 PMC7283827 32449791

[10] Wang H Li T Barbarino P Gauthier S Brodaty H Molinuevo JL Dementia care during COVID-19. Lancet (2020) 395(10231):1190–1. 10.1016/S0140-6736(20)30755-8 PMC7146671 32240625

[11] Bianchetti A Rozzini R Guerini F Boffelli S Ranieri P Minelli G Clinical presentation of COVID19 in dementia patients. J Nutr Health Aging (2020) 24(6):560–2. 10.1007/s12603-020-1389-1 PMC7227170 32510106

[12] Cheng ST Dementia caregiver burden: a research update and critical analysis. Curr Psychiatry Rep (2017) 19(9):64. 10.1007/s11920-017-0818-2 28795386 PMC5550537

[13] Edelman LS McConnell ES Kennerly SM Alderden J Horn SD Yap TL Mitigating the effects of a pandemic: facilitating improved nursing home care delivery through technology. JMIR Aging (2020) 3(1):e20110. 10.2196/20110 32412909 PMC7252197

[14] Holthe T Halvorsrud L Karterud D Hoel KA Lund A Usability and acceptability of technology for community-dwelling older adults with mild cognitive impairment and dementia: a systematic literature review. Clin Interv Aging (2018) 13:863–86. 10.2147/CIA.S154717 PMC5942395 29765211

[15] Canevelli M Bruno G Cesari M Providing simultaneous COVID-19-sensitive and dementia-sensitive care as we transition from crisis care to ongoing care. J Am Med Dir Assoc (2020) 21(7):968–9. 10.1016/j.jamda.2020.05.025 PMC7241363 32536553

[16] Moher D Liberati A Tetzlaff J Altman DG PRISMA Group Preferred reporting items for systematic reviews and meta-analyses: the PRISMA statement. Ann Intern Med (2009) 151(4):264–9, W64. 10.7326/0003-4819-151-4-200908180-00135 19622511

[17] Weinberg MS Patrick RE Schwab NA Owoyemi P May R McManus AJ Clinical trials and tribulations in the COVID-19 era. Am J Geriatr Psychiatry (2020) 28(9):913–20. 10.1016/j.jagp.2020.05.016 PMC7236727 32507686

[18] Gerritsen DL Oude Voshaar RC The effects of the COVID-19 virus on mental healthcare for older people in The Netherlands. Int Psychogeriatr (2020) 3:1–4. 10.1017/S1041610220001040 PMC7300185 32491980

[19] Isaia G Marinello R Tibaldi V Tamone C Bo M Atypical presentation of Covid-19 in an older adult with severe Alzheimer disease. Am J Geriatr Psychiatry (2020) 28(7):790–1. 10.1016/j.jagp.2020.04.018 PMC7175908 32381283

[20] Ousset PJ Vellas B Viewpoint: impact of the Covid-19 outbreak on the clinical and research activities of memory clinics: an Alzheimer’s disease center facing the Covid-19 crisis. J Prev Alzheimers Dis (2020) 7(3):197–8. 10.14283/jpad.2020.17 PMC7147199 32463074

[21] Rochford-Brennan H Keogh F Giving voice to those directly affected by the COVID-19 pandemic - the experience and reflections of a person with dementia. HRB Open Res (2020) 3:29. 10.12688/hrbopenres.13063.1 32518893 PMC7268151

[22] Padala SP Jendro AM Orr LC Facetime to reduce behavioral problems in a nursing home resident with Alzheimer’s dementia during COVID-19. Psychiatry Res (2020) 288:113028. 10.1016/j.psychres.2020.113028 32361337 PMC7177115

[23] Cipriani G Lucetti C Danti S Nuti A Apathy and dementia. Nosol Assess Manag J Nerv Ment Dis (2014) 202(10):718–24. 10.1097/NMD.0000000000000190 25265266

[24] Canevelli M Valletta M Toccaceli Blasi M Remoli G Sarti G Nuti F Facing dementia during the COVID-19 outbreak. J Am Geriatr Soc (2020) 68(8):1673–6. 10.1111/jgs.16644 PMC7300919 32516441

[25] Velayudhan L Aarsland D Ballard C Mental health of people living with dementia in care homes during COVID-19 pandemic. Int Psychogeriatr (2020), 1–2. 10.1017/S1041610220001088 PMC7302947 32487278

[26] Iaboni A Cockburn A Marcil M Rodrigues K Marshall C Garcia MA Achieving safe, effective, and compassionate quarantine or isolation of older adults with dementia in nursing homes. Am J Geriatr Psychiatry (2020) 28(8):835–8. 10.1016/j.jagp.2020.04.025 PMC7196899 32430111

[27] Kales HC Gitlin LN Lyketsos CG Assessment and management of behavioral and psychological symptoms of dementia. BMJ (2015) 350:h369. 10.1136/bmj.h369 25731881 PMC4707529

[28] Romeo R Zala D Knapp M Orrell M Fossey J Ballard C Improving the quality of life of care home residents with dementia: cost-effectiveness of an optimized intervention for residents with clinically significant agitation in dementia. Alzheimers Dement (2019) 15(2):282–91. 10.1016/j.jalz.2018.08.010 30470592

[29] Bostanciklioglu M Severe acute respiratory syndrome coronavirus 2 is penetrating to dementia research. Curr Neurovasc Res (2020) 17:1. 10.2174/1567202617666200522220509 32442082

[30] Shea YF Wan WH Chan MMK DeKosky ST Time-to-change: dementia care in COVID-19. Psychogeriatrics (2020) 1–2. 10.1111/psyg.12576 32510762

[31] Alzheimer’s Association Alzheimer’s disease facts and figures. Alzheimers Dement (2016) 12(4):459–509. 10.1016/j.jalz.2016.03.001 27570871

[32] Pachana N Beattie E Byrne G Brodaty H COVID-19 and psychogeriatrics: the view from Australia. Int Psychogeriatr (2020), 1–7. 10.1017/S1041610220000885 PMC7267101 32393404

[33] Phillips NA Chertkow H Pichora-Fuller MK Wittich W Special issues on using the Montreal Cognitive Assessment for telemedicine assessment during COVID-19. J Am Geriatr Soc (2020) 68(5):942–44. 10.1111/jgs.16469 32253754

[34] O’Shea E Remembering people with dementia during the COVID-19 crisis. HRB Open Res (2020) 3:15. 10.12688/hrbopenres.13030.2 32510035 PMC7195897

[35] Staal JA Sacks A Matheis R Collier L Calia T Hanif H The effects of Snoezelen (multi-sensory behavior therapy) and psychiatric care on agitation, apathy, and activities of daily living in dementia patients on a short term geriatric psychiatric inpatient unit. Int J Psychiatry Med (2007) 37(4):357–70. 10.2190/PM.37.4.a 18441625

[36] Brodaty H Arasaratnam C Meta-analysis of nonpharmacological interventions for neuropsychiatric symptoms of dementia. Am J Psychiatry (2012) 169(9):946–53. 10.1176/appi.ajp.2012.11101529 22952073

[37] Nowrangi MA Lyketsos CG Rosenberg PB Principles and management of neuropsychiatric symptoms in Alzheimer’s dementia. Alzheimers Res Ther (2015) 7(1):12. 10.1186/s13195-015-0096-3 27391771 PMC4571139

[38] Menon V Uddin LQ Saliency, switching, attention and control: a network model of insula function. Brain Struct Funct (2010) 214(5-6):655–67. 10.1007/s00429-010-0262-0 PMC2899886 20512370

[39] Trzepacz PT Yu P Bhamidipati PK Willis B Forrester T Tabas L Frontolimbic atrophy is associated with agitation and aggression in mild cognitive impairment and Alzheimer’s disease. Alzheimers Dement (2013) 9(5 Suppl):S95–S104.e1. 10.1016/j.jalz.2012.10.005 23253778 PMC3955297

[40] Bunn F Burn AM Goodman C Rait G Norton S Robinson L Comorbidity and dementia: a scoping review of the literature. BMC Med (2014) 12:192. 10.1186/s12916-014-0192-4 25358236 PMC4229610

[41] Marshall GA Fairbanks LA Tekin S Vinters HV Cummings JL Neuropathologic correlates of apathy in Alzheimer’s disease. Dement Geriatr Cognit Disord (2006) 21(3):144–7. 10.1159/000090674 16391476

[42] Starkstein SE Mizrahi R Capizzano AA Acion L Brockman S Power BD Neuroimaging correlates of apathy and depression in Alzheimer’s disease. J Neuropsychiatry Clin Neurosci (2009) 21(3):259–65. 10.1176/jnp.2009.21.3.259 19776304

[43] Geda YE Schneider LS Gitlin LN Miller DS Smith GS Bell J Neuropsychiatric symptoms in Alzheimer’s disease: past progress and anticipation of the future. Alzheimers Dement (2013) 9(5):602–8. 10.1016/j.jalz.2012.12.001 PMC3766403 23562430

[44] Livingston G Kelly L Lewis-Holmes E Baio G Morris S Patel N A systematic review of the clinical effectiveness and cost-effectiveness of sensory, psychological and behavioural interventions for managing agitation in older adults with dementia. Health Technol Assess (2014) 18(39)1–226, v-vi. 10.3310/hta18390 PMC4781145 24947468

[45] Gilmore-Bykovskyi A Block L Johnson R Goris ED Symptoms of apathy and passivity in dementia: a simultaneous concept analysis. J Clin Nurs (2019) 28(3-4):410–19. 10.1111/jocn.14663 PMC6326867 30184283

[46] Dyer SM Harrison SL Laver K Whitehead C Crotty M An overview of systematic reviews of pharmacological and non-pharmacological interventions for the treatment of behavioral and psychological symptoms of dementia. Int Psychogeriatr (2018) 30(3):295–309. 10.1017/S1041610217002344 29143695

[47] Chung JC Lai CK Chung PM French HP Snoezelen for dementia. Cochrane Database Syst Rev (2002) 4):CD003152. 10.1002/14651858.CD003152 PMC9002239 12519587

[48] Vink AC Birks JS Bruinsma MS Scholten RJ Music therapy for people with dementia. Cochrane Database Syst Rev (2004) 3):CD003477. 10.1002/14651858.CD003477.pub2 15266489

[49] Burns A Perry E Holmes C Francis P Morris J Howes MJ A double-blind placebo-controlled randomized trial of Melissa officinalis oil and donepezil for the treatment of agitation in Alzheimer’s disease. Dement Geriatr Cognit Disord (2011) 31(2):158–64. 10.1159/000324438 21335973

[50] O’Neil ME Freeman M Christensen V Telerant R Addleman A Kansagara D A systematic evidence review of non-pharmacological interventions for behavioral symptoms of dementia. Department of Veterans Affairs (US: Washington (DC (2011). 21634073

[51] Gitlin LN Winter L Dennis MP Hodgson N Hauck WW A biobehavioral home-based intervention and the well-being of patients with dementia and their caregivers: the COPE randomized trial. JAMA (2010) 304(9):983–91. 10.1001/jama.2010.1253 PMC4091681 20810376

[52] Nichols LO Martindale-Adams J Burns R Graney MJ Zuber J Translation of a dementia caregiver support program in a health care system–REACH VA. Arch Intern Med (2011) 171(4):353–9. 10.1001/archinternmed.2010.548 21357811

[53] Gitlin LN Corcoran M Winter L Boyce A Hauck WW A randomized, controlled trial of a home environmental intervention: effect on efficacy and upset in caregivers and on daily function of persons with dementia. Gerontologist (2001) 41(1):4–14. 10.1093/geront/41.1.4 11220813

[54] Gitlin LN Liebman J Winter L Are environmental interventions effective in the management of Alzheimer’s disease and related disorders?: A synthesis of the evidence. Alzheimers Care Today (2003) 4(2):85–107.

[55] Gitlin LN Belle SH Burgio LD Czaja SJ Mahoney D Gallagher-Thompson D Effect of multicomponent interventions on caregiver burden and depression: the REACH multisite initiative at 6-month follow-up. Psychol Aging (2003) 18(3):361–74. 10.1037/0882-7974.18.3.361 PMC2583061 14518800

[56] Abbasi J “Abandoned” nursing homes continue to face critical supply and staff shortages as COVID-19 toll has mounted. JAMA (2020) 324(2):123–5. 10.1001/jama.2020.10419 32525535

[57] Greenberg NE Wallick A Brown LM Impact of COVID-19 pandemic restrictions on community-dwelling caregivers and persons with dementia. Psychol Trauma (2020) 12(S1):S220–1. 10.1037/tra0000793 32584105

[58] Vaitheswaran S Lakshminarayanan M Ramanujam V Sargunan S Venkatesan S Experiences and needs of caregivers of persons with dementia in India during the COVID-19 pandemic-A qualitative study. Am J Geriatr Psychiatry (2020) 7:S1064–7481(20)30405-X. 10.1016/j.jagp.2020.06.026 PMC7340037 32736918

[59] Bjerre LM Farrell B Hogel M Graham L Lemay G McCarthy L Deprescribing antipsychotics for behavioural and psychological symptoms of dementia and insomnia: Evidence-based clinical practice guideline. Can Fam Physician (2018) 64(1):17–27. 29358245 PMC5962971

[60] Konovalov S Muralee S Tampi RR Anticonvulsants for the treatment of behavioral and psychological symptoms of dementia: a literature review. Int Psychogeriatr (2008) 20(2):293–308. 10.1017/S1041610207006540 18047764

[61] Ballard C Corbett A Management of neuropsychiatric symptoms in people with dementia. CNS Drugs (2010) 24(9):729–39. 10.2165/11319240-000000000-00000 20806986

[62] Kales HC Kim HM Zivin K Valenstein M Seyfried LS Chiang C Risk of mortality among individual antipsychotics in patients with dementia. Am J Psychiatry (2012) 169(1):71–9. 10.1176/appi.ajp.2011.11030347 PMC4269551 22193526

[63] Dols A Sienaert P van Gerven H Schouws S Stevens A Kupka R The prevalence and management of side effects of lithium and anticonvulsants as mood stabilizers in bipolar disorder from a clinical perspective: a review. Int Clin Psychopharmacol (2013) 28(6):287–96. 10.1097/YIC.0b013e32836435e2 23873292

[64] Liperoti R Onder G Landi F Lapane KL Mor V Bernabei R All-cause mortality associated with atypical and conventional antipsychotics among nursing home residents with dementia: a retrospective cohort study. J Clin Psychiatry (2009) 70(10):1340–7. 10.4088/JCP.08m04597yel PMC3775351 19906339

[65] Douglas IJ Smeeth L Exposure to antipsychotics and risk of stroke: self controlled case series study. BMJ (2008) 337:a1227. 10.1136/bmj.a1227 18755769 PMC2526549

[66] Gareri P De Fazio P Manfredi VG De Sarro G Use and safety of antipsychotics in behavioral disorders in elderly people with dementia. J Clin Psychopharmacol (2014) 34(1):109–23. 10.1097/JCP.0b013e3182a6096e 24158020

[67] Øksnebjerg L Woods B Vilsen CR Ruth K Gustafsson M Ringkøbing SP A Tablet App Supporting Self-Management for People With Dementia: Explorative Study of Adoption and Use Patterns. JMIR mHealth uHealth (2020) 8(1): e1469. 10.1080/13607863.2019.1625302 PMC6996756 31951217

[68] Vollmer Dahlke D Ory MG Emerging issues of intelligent assistive technology use among people with dementia and their caregivers: A U.S. Perspective. Front Public Health (2020) 8:191:191. 10.3389/fpubh.2020.00191 32528920 PMC7254691

[69] Cavallo F Aquilano M Arvati M An ambient assisted living approach in designing domiciliary services combined with innovative technologies for patients with Alzheimer’s disease: a case study. Am J Alzheimers Dis Other Demen (2015) 30(1):69–77. 10.1177/1533317514539724 24951634 PMC10852970

[70] Faucounau V Riguet M Orvoen G Lacombe A Rialle V Extra J Electronic tracking system and wandering in Alzheimer’s disease: a case study. Ann Phys Rehabil Med (2009) 52(7-8):579–87. 10.1016/j.rehab.2009.07.034 19744906

[71] Pot AM Willemse BM Horjus S A pilot study on the use of tracking technology: feasibility, acceptability, and benefits for people in early stages of dementia and their informal caregivers. Aging Ment Health (2012) 16(1):127–34. 10.1080/13607863.2011.596810 21780960

[72] Peek ST Wouters EJ van Hoof J Luijkx KG Boeije HR Vrijhoef HJ Factors influencing acceptance of technology for aging in place: a systematic review. Int J Med Inform (2014) 83(4):235–48. 10.1016/j.ijmedinf.2014.01.004 24529817

[73] Hascup ER Hascup KN Does SARS-CoV-2 infection cause chronic neurological complications? Geroscience (2020) 42(4):1083–7. 10.1007/s11357-020-00207-y PMC7247778 32451846

[74] Lozupone M La Montagna M Sardone R Seripa D Daniele A Panza F Can pharmacotherapy effectively reduce Alzheimer’s related agitation? Expert Opin Pharmacother (2020) 21(13):1517–22. 10.1080/14656566.2020.1770730 32475180

[75] Berman K Brodaty H Withall A Seeher K Pharmacologic treatment of apathy in dementia. Am J Geriatr Psychiatry (2012) 20(2):104–22. 10.1097/JGP.0b013e31822001a6 21841459

[76] Nowrangi MA Neuropsychiatric Aspects of Alzheimer Dementia: From Mechanism to Treatment. Psychiatr Clin North Am (2020) 43(2):383–97. 10.1016/j.psc.2020.02.012 32439028

[77] Lozupone M Panza F Piccininni M Copetti M Sardone R Imbimbo BP Social dysfunction in older age and relationships with cognition, depression, and apathy: the GreatAGE study. J Alzheimers Dis (2018) 65(3):989–1000. 10.3233/JAD-180466 30103335

