# Supplementary material for: Neuropsychiatric Symptoms in Elderly With Dementia During COVID-19 Pandemic: Definition, Treatment, and Future Directions
Source: Front Psychiatry. 2020 Sep 29;11:579842. doi: 10.3389/fpsyt.2020.579842 (PMC7550649; doi:10.3389/fpsyt.2020.579842)
Supplement: Supplementary file 1 [file Table_1.docx]

Supplement. Records resulting from our search conducted on June 19, 2020 with reasons for exclusion in the third column. All eligible studies are in bold.

| 1 | Kanberg N, Ashton NJ, Andersson LM, Yilmaz A, Lindh M, Nilsson S, Price RW, Blennow K, Zetterberg H, Gisslén M. Neurochemical evidence of astrocytic and neuronal injury commonly found in COVID-19. Neurology. 2020 Jun 16:10.1212/WNL.0000000000010111. doi: 10.1212/WNL.0000000000010111. Epub ahead of print. PMID: 32546655. | Unfocused |
| --- | --- | --- |
| 2 | Korczyn AD. Dementia in the COVID-19 Period. J Alzheimers Dis. 2020 Jun 8. doi: 10.3233/JAD-200609. Epub ahead of print. PMID: 32538858. | Editorial |
| 3 | Fotuhi M, Mian A, Meysami S, Raji CA. Neurobiology of COVID-19. J Alzheimers Dis. 2020 Jun 8. doi: 10.3233/JAD-200581. Epub ahead of print. PMID: 32538857. | Review |
| 4 | Benaque A, Gurruchaga MJ, Abdelnour C, Hernández I, Cañabate P, Alegret M, Rodríguez I, Rosende-Roca M, Tartari JP, Esteban E, López R, Gil S, Vargas L, Mauleón A, Espinosa A, Ortega G, Sanabria A, Pérez A, Alarcón E, González-Pérez A, Marquié M, Valero S, Tárraga L, Ruiz A, Boada M; Research Center and Memory Clinic, Fundació ACE. Dementia Care in Times of COVID-19: Experience at Fundació ACE in Barcelona, Spain. J Alzheimers Dis. 2020 Jun 12. doi: 10.3233/JAD-200547. Epub ahead of print. PMID: 32538856. | Unfocused |
| 5 | Naughton SX, Raval U, Pasinetti GM. Potential Novel Role of COVID-19 in Alzheimer's Disease and Preventative Mitigation Strategies. J Alzheimers Dis. 2020 Jun 12. doi: 10.3233/JAD-200537. Epub ahead of print. PMID: 32538855. | Comment |
| 6 | **Canevelli M, Bruno G, Cesari M. Providing Simultaneous COVID-19-sensitive and Dementia-Sensitive Care as We Transition from Crisis Care to Ongoing Care. J Am Med Dir Assoc. 2020 May 21:S1525-8610(20)30428-X. doi: 10.1016/j.jamda.2020.05.025. Epub ahead of print. PMID: 32536553; PMCID: PMC7241363.** | **Included** |
| 7 | Viel T, Chinta S, Rane A, Chamoli M, Buck H, Andersen J. Microdose lithium reduces cellular senescence in human astrocytes - a potential pharmacotherapy for COVID-19? Aging (Albany NY). 2020 Jun 13;12. doi: 10.18632/aging.103449. Epub ahead of print. PMID: 32534451. | In vitro |
| 8 | Blackman C, Farber S, Feifer RA, Mor V, White EM. An Illustration of SARS-CoV-2 Dissemination Within a Skilled Nursing Facility Using Heat Maps. J Am Geriatr Soc. 2020 Jun 13. doi: 10.1111/jgs.16642. Epub ahead of print. PMID: 32533847. | Unfocused |
| 9 | Beam CR, Kim AJ. Psychological sequelae of social isolation and loneliness might be a larger problem in young adults than older adults. Psychol Trauma. 2020 Jun 11. doi: 10.1037/tra0000774. Epub ahead of print. PMID: 32525372. | Unfocused |
| 10 | Cipriani G, Danti S, Nuti A, Carlesi C, Lucetti C, Di Fiorino M. A complication of coronavirus disease 2019: delirium. Acta Neurol Belg. 2020 Jun 10:1–6. doi: 10.1007/s13760-020-01401-7. Epub ahead of print. PMID: 32524537; PMCID: PMC7286634. | Review |
| 11 | **O'Shea E. Remembering people with dementia during the COVID-19 crisis. *HRB Open Res*. 2020;3:15. Published 2020 May 28. doi:10.12688/hrbopenres.13030.2** | **Included** |
| 12 | Castro-de-Araujo, Luís Fernando Silva, and Daiane Borges Machado. “Impact of COVID-19 on mental health in a Low and Middle-Income Country.” *Ciencia & saude coletiva* vol. 25,suppl 1 (2020): 2457-2460. doi:10.1590/1413-81232020256.1.10932020 | Unfocused |
| 13 | **Rochford-Brennan H, Keogh F. Giving voice to those directly affected by the COVID-19 pandemic - the experience and reflections of a person with dementia. HRB Open Res. 2020 May 28;3:29. doi: 10.12688/hrbopenres.13063.1. PMID: 32518893; PMCID: PMC7268151.** | **Included** |
| 14 | Suzuki M, Hotta M, Nagase A, Yamamoto Y, Hirakawa N, Satake Y, Nagata Y, Suehiro T, Kanemoto H, Yoshiyama K, Mori E, Hashimoto M, Ikeda M. The behavioral pattern of patients with frontotemporal dementia during the COVID-19 pandemic. Int Psychogeriatr. 2020 Jun 10:1-6. doi: 10.1017/S104161022000109X. Epub ahead of print. PMID: 32517839. | Unfocused |
| 15 | Covino M, De Matteis G, Santoro M, Sabia L, Simeoni B, Candelli M, Ojetti V, Franceschi F. Clinical characteristics and prognostic factors in COVID-19 patients aged ≥80 years. Geriatr Gerontol Int. 2020 Jun 9:10.1111/ggi.13960. doi: 10.1111/ggi.13960. Epub ahead of print. PMID: 32516861; PMCID: PMC7300699. | Unfocused |
| 16 | **Canevelli M, Valletta M, Toccaceli Blasi M, Remoli G, Sarti G, Nuti F, Sciancalepore F, Ruberti E, Cesari M, Bruno G. FACING DEMENTIA DURING THE COVID-19 OUTBREAK. J Am Geriatr Soc. 2020 Jun 9. doi: 10.1111/jgs.16644. Epub ahead of print. PMID: 32516441.** | **Included** |
| 17 | Nguyen LH, Drew DA, Joshi AD, Guo CG, Ma W, Mehta RS, Sikavi DR, Lo CH, Kwon S, Song M, Mucci LA, Stampfer MJ, Willett WC, Eliassen AH, Hart JE, Chavarro JE, Rich-Edwards JW, Davies R, Capdevila J, Lee KA, Lochlainn MN, Varsavsky T, Graham MS, Sudre CH, Cardoso MJ, Wolf J, Ourselin S, Steves CJ, Spector TD, Chan AT. Risk of COVID-19 among frontline healthcare workers and the general community: a prospective cohort study. Version 6. medRxiv [Preprint]. 2020 May 25:2020.04.29.20084111. doi: 10.1101/2020.04.29.20084111. PMID: 32511531; PMCID: PMC7273299. | Preprint |
| 18 | Shea YF, Wan WH, Chan MMK, DeKosky ST. Time-to-change: dementia care in COVID-19. Psychogeriatrics. 2020 Jun 8. doi: 10.1111/psyg.12576. Epub ahead of print. PMID: 32510762. | Note |
| **19** | **Bianchetti A, Rozzini R, Guerini F, Boffelli S, Ranieri P, Minelli G, Bianchetti L, Trabucchi M. Clinical Presentation of COVID19 in Dementia Patients. J Nutr Health Aging. 2020;24(6):560-562. doi: 10.1007/s12603-020-1389-1. PMID: 32510106.** | **Included** |
| 20 | **Weinberg MS, Patrick RE, Schwab NA, Owoyemi P, May R, McManus AJ, Gerber J, Harper DG, Arnold SE, Forester B. Clinical Trials and Tribulations in the COVID-19 Era. Am J Geriatr Psychiatry. 2020 May 19:S1064-7481(20)30345-6. doi: 10.1016/j.jagp.2020.05.016. Epub ahead of print. PMID: 32507686.** | **Included** |
| 21 | Palmieri L, Vanacore N, Donfrancesco C, Lo Noce C, Canevelli M, Punzo O, Raparelli V, Pezzotti P, Riccardo F, Bella A, Fabiani M, D'Ancona FP, Vaianella L, Tiple D, Colaizzo E, Palmer K, Rezza G, Piccioli A, Brusaferro S, Onder G; Italian National Institute of Health COVID-19 mortality group. Clinical Characteristics of Hospitalized Individuals Dying with COVID-19 by Age Group in Italy. J Gerontol A Biol Sci Med Sci. 2020 Jun 7:glaa146. doi: 10.1093/gerona/glaa146. Epub ahead of print. PMID: 32506122. | Unfocused |
| 22 | Graham N, Junghans C, Downes R, Sendall C, Lai H, McKirdy A, Elliott P, Howard R, Wingfield D, Priestman M, Ciechonska M, Cameron L, Storch M, Crone MA, Freemont PS, Randell P, McLaren R, Lang N, Ladhani S, Sanderson F, Sharp DJ. SARS-CoV-2 infection, clinical features and outcome of COVID-19 in United Kingdom nursing homes. J Infect. 2020 Jun 3:S0163-4453(20)30348-0. doi: 10.1016/j.jinf.2020.05.073. Epub ahead of print. PMID: 32504743. | Unfocused |
| 23 | Gomez-Pinedo U, Matias-Guiu J, Sanclemente-Alaman I, Moreno-Jimenez L, Montero-Escribano P. SARS-CoV2 as a potential trigger of neurodegenerative diseases [published online ahead of print, 2020 Jun 5]. *Mov Disord*. 2020;10.1002/mds.28179. doi:10.1002/mds.28179 | Unfocused |
| 24 | Kuper H, Banks LM, Bright T, Davey C, Shakespeare T. Disability-inclusive COVID-19 response: What it is, why it is important and what we can learn from the United Kingdom's response. Wellcome Open Res. 2020 Apr 28;5:79. doi: 10.12688/wellcomeopenres.15833.1. PMID: 32500099; PMCID: PMC7236579. | Unfocused |
| 25 | Heneka MT, Golenbock D, Latz E, Morgan D, Brown R. Immediate and long-term consequences of COVID-19 infections for the development of neurological disease. Alzheimers Res Ther. 2020 Jun 4;12(1):69. doi: 10.1186/s13195-020-00640-3. PMID: 32498691; PMCID: PMC7271826. | Unfocused |
| 26 | Bersano A, Kraemer M, Touzé E, Weber R, Alamowitch S, Sibon I, Pantoni L. Stroke care during the Covid-19 pandemic: Experience from three large European countries. Eur J Neurol. 2020 Jun 3. doi: 10.1111/ene.14375. Epub ahead of print. PMID: 32492764. | Review |
| 27 | **Gerritsen DL, Oude Voshaar RC. The effects of the COVID-19 virus on mental healthcare for older people in The Netherlands. Int Psychogeriatr. 2020 Jun 3:1-10. doi: 10.1017/S1041610220001040. Epub ahead of print. PMID: 32491980.** | **Included** |
| 28 | Lahiri D, Ardila A. COVID-19 Pandemic: A Neurological Perspective. Cureus. 2020 Apr 29;12(4):e7889. doi: 10.7759/cureus.7889. PMID: 32489743; PMCID: PMC7255551. | Review |
| 29 | Chong TWH, Curran E, Ames D, Lautenschlager NT, Castle DJ. Mental health of older adults during the COVID-19 pandemic: lessons from history to guide our future. Int Psychogeriatr. 2020 Jun 3:1-4. doi: 10.1017/S1041610220001003. Epub ahead of print. PMID: 32489166. | Editorial |
| 30 | **Velayudhan L, Aarsland D, Ballard C. Mental health of people living with dementia in care homes during COVID-19 pandemic. Int Psychogeriatr. 2020 Jun 3:1-5. doi: 10.1017/S1041610220001088. Epub ahead of print. PMID: 32487278.** | **Included** |
| 31 | Funders' Efforts: Aging And Health, COVID-19. *Health Aff (Millwood)*. 2020;39(6):1092‐1093. doi:10.1377/hlthaff.2020.00599 | Unfocused |
| 32 | Geerts H, van der Graaf PH. Salvaging CNS Clinical Trials halted due to COVID-19. CPT Pharmacometrics Syst Pharmacol. 2020 May 28. doi: 10.1002/psp4.12535. Epub ahead of print. PMID: 32468710. | Unfocused |
| 33 | Busetto L, Bettini S, Fabris R, Serra R, Dal Pra' C, Maffei P, Rossato M, Fioretto P, Vettor R. Obesity and COVID-19: an Italian snapshot. Obesity (Silver Spring). 2020 May 28:10.1002/oby.22918. doi: 10.1002/oby.22918. Epub ahead of print. PMID: 32463545; PMCID: PMC7283686. | Unfocused |
| 34 | **Ousset PJ, Vellas B. Viewpoint: Impact of the Covid-19 Outbreak on the Clinical and Research Activities of Memory Clinics: An Alzheimer's Disease Center Facing the Covid-19 Crisis. J Prev Alzheimers Dis. 2020;7(3):197-198. doi: 10.14283/jpad.2020.17. PMID: 32463074; PMCID: PMC7147199.** | **Included** |
| 35 | Palmer K, Monaco A, Kivipelto M, Onder G, Maggi S, Michel JP, Prieto R, Sykara G, Donde S. The potential long-term impact of the COVID-19 outbreak on patients with non-communicable diseases in Europe: consequences for healthy ageing. Aging Clin Exp Res. 2020 May 26:1–6. doi: 10.1007/s40520-020-01601-4. Epub ahead of print. PMID: 32458356; PMCID: PMC7248450. | Unfocused |
| 36 | Hascup ER, Hascup KN. Does SARS-CoV-2 infection cause chronic neurological complications? Geroscience. 2020 May 25:1–5. doi: 10.1007/s11357-020-00207-y. Epub ahead of print. PMID: 32451846; PMCID: PMC7247778. | Unfocused |
| 37 | **Beatriz Lara B, Carnes A, Dakterzada F, Benitez I, Piñol-Ripoll G. Neuropsychiatric symptoms and quality of life in Spanish Alzheimer's disease patients during COVID-19 lockdown. Eur J Neurol. 2020 May 25. doi: 10.1111/ene.14339. Epub ahead of print. PMID: 32449791.** | **Included** |
| 38 | Frontera J, Mainali S, Fink EL, Robertson CL, Schober M, Ziai W, Menon D, Kochanek PM, Suarez JI, Helbok R, McNett M, Chou SH; GCS-NeuroCOVID Study. Global Consortium Study of Neurological Dysfunction in COVID-19 (GCS-NeuroCOVID): Study Design and Rationale. Neurocrit Care. 2020 May 22:1–10. doi: 10.1007/s12028-020-00995-3. Epub ahead of print. PMID: 32445105; PMCID: PMC7243953. | Rationale |
| 39 | **Bostanciklioglu M. Severe acute respiratory syndrome coronavirus 2 is penetrating to dementia research [published online ahead of print, 2020 May 22]. *Curr Neurovasc Res*. 2020;10.2174/1567202617666200522220509. doi:10.2174/1567202617666200522220509** | **Included** |
| 40 | **Iaboni A, Cockburn A, Marcil M, Rodrigues K, Marshall C, Garcia MA, Quirt H, Reynolds KB, Keren R, Flint AJ. Achieving Safe, Effective, and Compassionate Quarantine or Isolation of Older Adults With Dementia in Nursing Homes. Am J Geriatr Psychiatry. 2020 May 4:S1064-7481(20)30326-2. doi: 10.1016/j.jagp.2020.04.025. Epub ahead of print. PMID: 32430111; PMCID: PMC7196899.** | **Included** |
| 41 | Ramón Martínez Riera J, Gras-Nieto E. ATENCIÓN DOMICILIARIA Y COVID-19. ANTES, DURANTE Y DESPUÉS DEL ESTADO DE ALARMA [HOME CARE AND COVID-19. BEFORE, IN AND AFTER THE STATE OF ALARM]. Enferm Clin. 2020 May 15. Spanish. doi: 10.1016/j.enfcli.2020.05.003. Epub ahead of print. PMID: 32419772; PMCID: PMC7225710. | Non-English |
| 42 | Mahajan N, Singla M, Singh B, Sajja V, Bansal P, Paul B, Goel P, Midha R, Bansal R, Singh G. 2019-NCoV: What Every Neurologist Should Know? Ann Indian Acad Neurol. 2020 Apr;23(Suppl 1):S28-S32. doi: 10.4103/aian.AIAN_210_20. Epub 2020 Apr 17. PMID: 32419751; PMCID: PMC7213032. | Review |
| 43 | Pilotto A, Odolini S, Stefano Masciocchi S, Comelli A, Volonghi I, Gazzina S, Nocivelli S, Pezzini A, Focà E, Caruso A, Leonardi M, Pasolini MP, Roberto Gasparotti R, Francesco Castelli F, Ashton NJ, Blennow K, Zetterberg H, Padovani A. Steroid-responsive encephalitis in Covid-19 disease. Ann Neurol. 2020 May 17. doi: 10.1002/ana.25783. Epub ahead of print. PMID: 32418288 | Unfocused |
| 44 | Coen M, Allali G, Adler D, Serratrice J. Hypoxemia in COVID-19; Comment on: "The neuroinvasive potential of SARS - CoV2 may play a role in the respiratory failure of COVID-19 patients" [published online ahead of print, 2020 May 17]. *J Med Virol*. 2020;10.1002/jmv.26020. | Comment |
| 45 | Ng Kee Kwong KC, Mehta PR, Shukla G, Mehta AR. COVID-19, SARS and MERS: A neurological perspective [published online ahead of print, 2020 May 5]. *J Clin Neurosci*. 2020;S0967-5868(20)31185-1. doi:10.1016/j.jocn.2020.04.124 | Unfocused |
| 46 | **Edelman LS, McConnell ES, Kennerly SM, Alderden J, Horn SD, Yap TL. Mitigating the Effects of a Pandemic: Facilitating Improved Nursing Home Care Delivery Through Technology. *JMIR Aging*. 2020;3(1):e20110. Published 2020 May 26. doi:10.2196/20110** | **Included** |
| 47 | Zhu J, Sun L, Zhang L, Wang H, Fan A, Yang B, Li W, Xiao S. Prevalence and Influencing Factors of Anxiety and Depression Symptoms in the First-Line Medical Staff Fighting Against COVID-19 in Gansu. Front Psychiatry. 2020 Apr 29;11:386. doi: 10.3389/fpsyt.2020.00386. PMID: 32411034; PMCID: PMC7202136. | Unfocused |
| 48 | Leocani L, Diserens K, Moccia M, Caltagirone C. Disability through COVID-19 pandemic: Neurorehabilitation cannot wait [published online ahead of print, 2020 May 13]. *Eur J Neurol*. 2020;10.1111/ene.14320. doi:10.1111/ene.14320 | Letter |
| 49 | **Goodman-Casanova JM, Dura-Perez E, Guzman-Parra J, Cuesta-Vargas A, Mayoral-Cleries F. Telehealth Home Support During COVID-19 Confinement for Community-Dwelling Older Adults With Mild Cognitive Impairment or Mild Dementia: Survey Study. *J Med Internet Res*. 2020;22(5):e19434. Published 2020 May 22. doi:10.2196/19434** | **Included** |
| 50 | Klok FA, Boon GJAM, Barco S, Endres M, Geelhoed JJM, Knauss S, Rezek SA, Spruit MA, Vehreschild J, Siegerink B. The Post-COVID-19 Functional Status (PCFS) Scale: a tool to measure functional status over time after COVID-19. Eur Respir J. 2020 May 12:2001494. doi: 10.1183/13993003.01494-2020. Epub ahead of print. PMID: 32398306; PMCID: PMC7236834. | Unfocused |
| 51 | Menni C, Valdes AM, Freidin MB, Sudre CH, Nguyen LH, Drew DA, Ganesh S, Varsavsky T, Cardoso MJ, El-Sayed Moustafa JS, Visconti A, Hysi P, Bowyer RCE, Mangino M, Falchi M, Wolf J, Ourselin S, Chan AT, Steves CJ, Spector TD. Real-time tracking of self-reported symptoms to predict potential COVID-19. Nat Med. 2020 May 11. doi: 10.1038/s41591-020-0916-2. Epub ahead of print. PMID: 32393804. | Unfocused |
| 52 | **Pachana NA, Beattie E, Byrne GJ, Brodaty H. COVID-19 and psychogeriatrics: the view from Australia [published online ahead of print, 2020 May 12]. *Int Psychogeriatr*. 2020;1‐7. doi:10.1017/S1041610220000885** | **Included** |
| 53 | Clarfield AM, Dwolatzky T, Brill S, Press Y, Glick S, Shvartzman P, Doron II. Israel Ad Hoc COVID-19 Committee: Guidelines for Care of Older Persons During a Pandemic. J Am Geriatr Soc. 2020 May 11:10.1111/jgs.16554. doi: 10.1111/jgs.16554. Epub ahead of print. PMID: 32392624; PMCID: PMC7272988. | Unfocused |
| 54 | Valenzuela PL, Santos-Lozano A, Lista S, Serra-Rexach JA, Emanuele E, Lucia A. Coronavirus Lockdown: Forced Inactivity for the Oldest Old? [published online ahead of print, 2020 Apr 28]. *J Am Med Dir Assoc*. 2020;S1525-8610(20)30285-1. doi:10.1016/j.jamda.2020.03.026 | Unfocused |
| 55 | **Isaia G, Marinello R, Tibaldi V, Tamone C, Bo M. Atypical Presentation of Covid-19 in an Older Adult With Severe Alzheimer Disease [published online ahead of print, 2020 Apr 22]. *Am J Geriatr Psychiatry*. 2020;S1064-7481(20)30319-5. doi:10.1016/j.jagp.2020.04.018** | **Included** |
| 56 | **Padala SP, Jendro AM, Orr LC. Facetime to reduce behavioral problems in a nursing home resident with Alzheimer's dementia during COVID-19. *Psychiatry Res*. 2020;288:113028. doi:10.1016/j.psychres.2020.113028** | **Included** |
| 57 | Mani Mishra P, Uversky VN, Nandi CK. Serum albumin-mediated strategy for the effective targeting of SARS-CoV-2 [published online ahead of print, 2020 Apr 24]. *Med Hypotheses*. 2020;140:109790. doi:10.1016/j.mehy.2020.109790 | Unfocused |
| 58 | Abbatecola AM, Antonelli-Incalzi R. Editorial: COVID-19 Spiraling of Frailty in Older Italian Patients. *J Nutr Health Aging*. 2020;24(5):453‐455. doi:10.1007/s12603-020-1357-9 | Editorial |
| 59 | Cai M, Wang G, Zhang L, Gao J, Xia Z, Zhang P, Wang Z, Cai K, Wang G, Tao K. Performing abdominal surgery during the COVID-19 epidemic in Wuhan, China: a single-centred, retrospective, observational study. Br J Surg. 2020 Jun;107(7):e183-e185. doi: 10.1002/bjs.11643. Epub 2020 Apr 27. PMID: 32339259; PMCID: PMC7267650. | Unfocused |
| 60 | Zimmerman S, Sloane PD, Katz PR, Kunze M, O'Neil K, Resnick B. The Need to Include Assisted Living in Responding to the COVID-19 Pandemic. *J Am Med Dir Assoc*. 2020;21(5):572‐575. doi:10.1016/j.jamda.2020.03.024 | Editorial |
| 61 | **Brown EE, Kumar S, Rajji TK, Pollock BG, Mulsant BH. Anticipating and Mitigating the Impact of the COVID-19 Pandemic on Alzheimer's Disease and Related Dementias [published online ahead of print, 2020 Apr 18]. *Am J Geriatr Psychiatry*. 2020;S1064-7481(20)30294-3.** | **Included** |
| 62 | Cipriani G, Fiorino MD. Access to Care for Dementia Patients Suffering From COVID-19 [published online ahead of print, 2020 Apr 17]. *Am J Geriatr Psychiatry*. 2020;S1064-7481(20)30293-1. doi:10.1016/j.jagp.2020.04.009 | Unfocused |
| 63 | Butler MJ, Barrientos RM. The impact of nutrition on COVID-19 susceptibility and long-term consequences. Brain Behav Immun. 2020 Apr18:S0889-1591(20)30537-7. doi: 10.1016/j.bbi.2020.04.040. Epub ahead of print.PMID: 32311498; PMCID: PMC7165103 | Unfocused |
| 64 | Cawthon P, Orwoll E, Ensrud K, Cauley JA, Kritchevsky SB, Cummings SR, Newman A. Assessing the impact of the covid-19 pandemic and accompanying mitigation efforts on older adults. J Gerontol A Biol Sci Med Sci. 2020 Apr20:glaa099. doi: 10.1093/gerona/glaa099. Epub ahead of print. PMID: 32307522; PMCID: PMC7188163. | Letter |
| 65 | Huang HT, Chen TC, Liu TY, Chiu CF, Hsieh WC, Yang CJ, Chen YH. How to prevent outbreak of a hospital-affiliated dementia day-care facility in the pandemic COVID-19 infection in Taiwan. J Microbiol Immunol Infect. 2020 Jun;53(3):394-395. doi: 10.1016/j.jmii.2020.04.007. Epub 2020 Apr 13. PMID: 32305270; PMCID: PMC7152916. | Letter |
| 66 | Holmes EA, O'Connor RC, Perry VH, Tracey I, Wessely S, Arseneault L, Ballard C, Christensen H, Cohen Silver R, Everall I, Ford T, John A, Kabir T, King K, Madan I, Michie S, Przybylski AK, Shafran R, Sweeney A, Worthman CM, Yardley L, Cowan K, Cope C, Hotopf M, Bullmore E. Multidisciplinary research priorities for the COVID-19 pandemic: a call for action for mental health science. Lancet Psychiatry. 2020 Jun;7(6):547-560. doi: 10.1016/S2215-0366(20)30168-1. Epub 2020 Apr 15. PMID: 32304649; PMCID: PMC7159850. | Unfocused |
| 67 | Myers L, Balakrishnan S, Reddy S, Gholamrezanezhad A. Coronavirus Outbreak: Is Radiology Ready? Mass Casualty Incident Planning. J Am Coll Radiol. 2020 Jun;17(6):724-729. doi: 10.1016/j.jacr.2020.03.025. Epub 2020 Apr 3. PMID: 32304643; PMCID: PMC7128279 | Unfocused |
| 68 | Coccolini F, Sartelli M, Kluger Y, Pikoulis E, Karamagioli E, Moore EE, Biffl WL, Peitzman A, Hecker A, Chirica M, Damaskos D, Ordonez C, Vega F, Fraga GP, Chiarugi M, Di Saverio S, Kirkpatrick AW, Abu-Zidan F, Mefire AC, Leppaniemi A, Khokha V, Sakakushev B, Catena R, Coimbra R, Ansaloni L, Corbella D, Catena F. COVID-19 the showdown for mass casualty preparedness and management: the Cassandra Syndrome. Version 2. World J Emerg Surg. 2020 Apr 9;15(1):26. doi: 10.1186/s13017-020-00304-5. PMID: 32272957; PMCID: PMC7145275 | Unfocused |
| 69 | Wang H, Li T, Gauthier S, Yu E, Tang Y, Barbarino P, Yu X. Coronavirus epidemic and geriatric mental healthcare in China: how a coordinated response by professional organizations helped older adults during an unprecedented crisis. Int Psychogeriatr. 2020 Apr 9:1-4. doi: 10.1017/S1041610220000551. Epub ahead of print. PMID: 32268928; PMCID: PMC7184143 | Comment |
| 70 | **Phillips NA, Chertkow H, Pichora-Fuller MK, Wittich W. Special Issues on Using the Montreal Cognitive Assessment for telemedicine Assessment During COVID-19. J Am Geriatr Soc. 2020 May;68(5):942-944. doi: 10.1111/jgs.16469. Epub 2020 Apr 15. PMID: 32253754** | **Included** |
| 71 | **Wang H, Li T, Barbarino P, Gauthier S, Brodaty H, Molinuevo JL, Xie H, Sun Y, Yu E, Tang Y, Weidner W, Yu X. Dementia care during COVID-19. Lancet. 2020 Apr 11;395(10231):1190-1191. doi: 10.1016/S0140-6736(20)30755-8. Epub 2020 Mar 30. PMID: 32240625; PMCID: PMC7146671** | **Included** |
| 71 | Alzheimer's Disease Research Enterprise in the Era of COVID-19/SARS-CoV-2. Alzheimers Dement. 2020 Apr;16(4):587-588. doi: 10.1002/alz.12093. Epub 2020 Mar 22. PMID: 32202041; PMCID: PMC7161893 | Editorial |
| 73 | Deiner S, Fleisher LA, Leung JM, Peden C, Miller T, Neuman MD; ASA Committee on Geriatric Anesthesia and the ASA Perioperative Brain Health Initiative. Adherence to recommended practices for perioperative anesthesia care for older adults among US anesthesiologists: results from the ASA Committee on Geriatric Anesthesia-Perioperative Brain Health Initiative ASA member survey. Perioper Med (Lond). 2020 Feb 25;9:6. doi: 10.1186/s13741-020-0136-9. PMID: 32123562; PMCID: PMC7041201 | Unfocused |
| 74 | Jagodzinski A, Johansen C, Koch-Gromus U, Aarabi G, Adam G, Anders S, Augustin M, der Kellen RB, Beikler T, Behrendt CA, Betz CS, Bokemeyer C, Borof K, Briken P, Busch CJ, Büchel C, Brassen S, Debus ES, Eggers L, Fiehler J, Gallinat J, Gellißen S, Gerloff C, Girdauskas E, Gosau M, Graefen M, Härter M, Harth V, Heidemann C, Heydecke G, Huber TB, Hussein Y, Kampf MO, von dem Knesebeck O, Konnopka A, König HH, Kromer R, Kubisch C, Kühn S, Loges S, Löwe B, Lund G, Meyer C, Nagel L, Nienhaus A, Pantel K, Petersen E, Püschel K, Reichenspurner H, Sauter G, Scherer M, Scherschel K, Schiffner U, Schnabel RB, Schulz H, Smeets R, Sokalskis V, Spitzer MS, Terschüren C, Thederan I, Thoma T, Thomalla G, Waschki B, Wegscheider K, Wenzel JP, Wiese S, Zyriax BC, Zeller T, Blankenberg S. Rationale and Design of the Hamburg City Health Study. Eur J Epidemiol. 2020 Feb;35(2):169-181. doi: 10.100 | Unfocused |
| 75 | Walker LAS, Lindsay-Brown AP, Berard JA. Cognitive Fatigability Interventions in Neurological Conditions: A Systematic Review. Neurol Ther. 2019 Dec;8(2):251-271. doi: 10.1007/s40120-019-00158-3. Epub 2019 Oct 4. PMID: 31586303; PMCID: PMC6858900 | Review |
| 76 | Romero-Ayuso D, Castillero-Perea Á, González P, Navarro E, Molina-Massó JP, Funes MJ, Ariza-Vega P, Toledano-González A, Triviño-Juárez JM. Assessment of cognitive instrumental activities of daily living: a systematic review. Disabil Rehabil. 2019 Sep 24:1-17. doi: 10.1080/09638288.2019.1665720. Epub ahead of print. PMID: 31549907 | Review |
| 77 | Knaak C, Vorderwülbecke G, Spies C, Piper SK, Hadzidiakos D, Borchers F, Brockhaus WR, Radtke FM, Lachmann G. C-reactive protein for risk prediction of post-operative delirium and post-operative neurocognitive disorder. Acta Anaesthesiol Scand. 2019 Nov;63(10):1282-1289. doi: 10.1111/aas.13441. Epub 2019 Jul 29. PMID: 31283835 | Unfocused |
| 78 | Malsch C, Liman T, Wiedmann S, Siegerink B, Georgakis MK, Tiedt S, Endres M, Heuschmann PU. Outcome after stroke attributable to baseline factors-The PROSpective Cohort with Incident Stroke (PROSCIS). PLoS One. 2018 Sep 26;13(9):e0204285. doi: 10.1371/journal.pone.0204285. PMID: 30256828; PMCID: PMC6157870 | Unfocused |
| 79 | Cassano P, Dording C, Thomas G, Foster S, Yeung A, Uchida M, Hamblin MR, Bui E, Fava M, Mischoulon D, Iosifescu DV. Effects of transcranial photobiomodulation with near-infrared light on sexual dysfunction. Lasers Surg Med. 2019 Feb;51(2):127-135. doi: 10.1002/lsm.23011. Epub 2018 Sep 17. PMID: 30221776; PMCID: PMC6382556 | Unfocused |
| 80 | Mundi MS, Patel J, McClave SA, Hurt RT. Current perspective for tube feeding in the elderly: from identifying malnutrition to providing of enteral nutrition. Clin Interv Aging. 2018 Aug 1;13:1353-1364. doi: 10.2147/CIA.S134919. PMID: 30122907; PMCID: PMC6080667 | Unfocused |
| 81 | Mrkobrada M, Chan MTV, Cowan D, Spence J, Campbell D, Wang CY, Torres D, Malaga G, Sanders RD, Brown C, Sigamani A, Szczeklik W, Dmytriw AA, Agid R, Smith EE, Hill MD, Sharma M, Sharma M, Tsai S, Mensinkai A, Sahlas DJ, Guyatt G, Pettit S, Copland I, Wu WKK, Yu SCH, Gin T, Loh PS, Ramli N, Siow YL, Short TG, Waymouth E, Kumar J, Dasgupta M, Murkin JM, Fuentes M, Ortiz-Soriano V, Lindroth H, Simpson S, Sessler D, Devereaux PJ. Rationale and design for the detection and neurological impact of cerebrovascular events in non-cardiac surgery patients cohort evaluation (NeuroVISION) study: a prospective international cohort study. BMJ Open. 2018 Jul 6;8(7):e021521. doi: 10.1136/bmjopen-2018-021521. PMID: 29982215; PMCID: PMC6042543 | Unfocused |
| 82 | Deiner S, Luo X, Lin HM, Sessler DI, Saager L, Sieber FE, Lee HB, Sano M; and the Dexlirium Writing Group, Jankowski C, Bergese SD, Candiotti K, Flaherty JH, Arora H, Shander A, Rock P. Intraoperative Infusion of Dexmedetomidine for Prevention of Postoperative Delirium and Cognitive Dysfunction in Elderly Patients Undergoing Major Elective Noncardiac Surgery: A Randomized Clinical Trial. JAMA Surg. 2017 Aug 16;152(8):e171505. doi: 10.1001/jamasurg.2017.1505. Epub 2017 Aug 16. PMID: 28593326; PMCID: PMC5831461 | Unfocused |
| 83 | Dekeyzer S, De Kock I, Nikoubashman O, Vanden Bossche S, Van Eetvelde R, De Groote J, Acou M, Wiesmann M, Deblaere K, Achten E. "Unforgettable" - a pictorial essay on anatomy and pathology of the hippocampus. Insights Imaging. 2017 Apr;8(2):199-212. doi: 10.1007/s13244-016-0541-2. Epub 2017 Jan 20. PMID: 28108955; PMCID: PMC5359145 | Unfocused |
| 84 | Bateman RM et al. 36th International Symposium on Intensive Care and Emergency Medicine: Brussels, Belgium. 15-18 March 2016. Crit Care.2016 Apr 20;20(Suppl 2):94. doi: 10.1186/s13054-016-1208-6. Erratum in: Crit Care. 2016 Oct 24;20:347. PMID: 27885969; PMCID: PMC5493079 | Unfocused |
| 85 | Bernbaum M, Menon BK, Fick G, Smith EE, Goyal M, Frayne R, Coutts SB. Reduced blood flow in normal white matter predicts development of leukoaraiosis. J Cereb Blood Flow Metab. 2015 Oct;35(10):1610-5. doi: 10.1038/jcbfm.2015.92. Epub 2015 May 13. PMID: 25966951; PMCID: PMC4640308 | Unfocused |
| 86 | Ghosh A, Pithadia AS, Bhat J, Bera S, Midya A, Fierke CA, Ramamoorthy A, Bhunia A. Self-assembly of a nine-residue amyloid-forming peptide fragment of SARS corona virus E-protein: mechanism of self aggregation and amyloid- inhibition of hIAPP. Biochemistry. 2015 Apr 7;54(13):2249-2261. doi: 10.1021/acs.biochem.5b00061. Epub 2015 Mar 24. PMID: 25785896; PMCID: PMC4903029 | Unfocused |
| 87 | Giannitti F, Diab S, Mete A, Stanton JB, Fielding L, Crossley B, Sverlow K, Fish S, Mapes S, Scott L, Pusterla N. Necrotizing Enteritis and Hyperammonemic Encephalopathy Associated With Equine Coronavirus Infection in Equids. Vet Pathol. 2015 Nov;52(6):1148-56. doi: 10.1177/0300985814568683. Epub 2015 Feb 3. PMID: 25648965 | Unfocused |
| 88 | Lamarre NS, Braverman AS, Malykhina AP, Barbe MF, Ruggieri MR Sr. Alterations in nerve-evoked bladder contractions in a coronavirus-induced mouse model of multiple sclerosis. PLoS One. 2014 Oct 13;9(10):e109314. doi: 10.1371/journal.pone.0109314. PMID: 25310403; PMCID: PMC4195612 | Unfocused |
| 89 | Deiner S, Chu I, Mahanian M, Lin HM, Hecht AC, Silverstein JH. Prone position is associated with mild cerebral oxygen desaturation in elderly surgical patients. PLoS One. 2014 Sep 12;9(9):e106387. doi: 10.1371/journal.pone.0106387. PMID: 25216265; PMCID: PMC4162535 | Unfocused |
| 90 | Brison E, Jacomy H, Desforges M, Talbot PJ. Novel treatment with neuroprotective and antiviral properties against a neuroinvasive human respiratory virus. J Virol. 2014 Feb;88(3):1548-63. doi: 10.1128/JVI.02972-13. Epub 2013 Nov 13. PMID: 24227863; PMCID: PMC3911624 | Unfocused |
| 91 | Levros LC Jr, Labrie M, Charfi C, Rassart E. Binding and repressive activities of apolipoprotein E3 and E4 isoforms on the human ApoD promoter. Mol Neurobiol. 2013 Dec;48(3):669-80. doi: 10.1007/s12035-013-8456-0. Epub 2013 May 30. PMID: 23715769; PMCID: PMC7090986 | Unfocused |
| 92 | Nyer M, Farabaugh A, Fehling K, Soskin D, Holt D, Papakostas GI, Pedrelli P, Fava M, Pisoni A, Vitolo O, Mischoulon D. Relationship between sleep disturbance and depression, anxiety, and functioning in college students. Depress Anxiety. 2013 Sep;30(9):873-80. doi: 10.1002/da.22064. Epub 2013 May 16. PMID: 23681944; PMCID: PMC3791314 | Unfocused |
| 93 | Ballard C, Jones E, Gauge N, Aarsland D, Nilsen OB, Saxby BK, Lowery D, Corbett A, Wesnes K, Katsaiti E, Arden J, Amoako D, Prophet N, Purushothaman B, Green D. Optimised anaesthesia to reduce post operative cognitive decline (POCD) in older patients undergoing elective surgery, a randomised controlled trial. PLoS One. 2012;7(6):e37410. doi: 10.1371/journal.pone.0037410. Epub 2012 Jun 15. Erratum in: PLoS One. 2012;7(9). doi:10.1371/annotation/1cc38e55-23e8-44a5-ac2b-43c7b2a880f9. Amaoko, Derek [corrected to Amoako, Derek]. Erratum in: PLoS One. 2013;8(9). doi:10.1371/annotation | Unfocused |
| 94 | Sy M, Kitazawa M, Medeiros R, Whitman L, Cheng D, Lane TE, Laferla FM. Inflammation induced by infection potentiates tau pathological features in transgenic mice. Am J Pathol. 2011 Jun;178(6):2811-22. doi: 10.1016/j.ajpath.2011.02.012. Epub 2011 Apr 30. PMID: 21531375; PMCID: PMC3124234 | Unfocused |
| 95 | Do Carmo S, Jacomy H, Talbot PJ, Rassart E. Neuroprotective effect of apolipoprotein D against human coronavirus OC43-induced encephalitis in mice. J Neurosci. 2008 Oct 8;28(41):10330-8. doi: 10.1523/JNEUROSCI.2644-08.2008. PMID: 18842892; PMCID: PMC6671015. | Unfocused |
| 96 | Power J. Old bugs and new: classical and emerging pathogens--relevance to dental practice. J Ir Dent Assoc. 2004 Summer;50(2):79-80. PMID: 15239585 | Unfocused |
| 97 | Cristallo A, Gambaro F, Biamonti G, Ferrante P, Battaglia M, Cereda PM. Human coronavirus polyadenylated RNA sequences in cerebrospinal fluid from multiple sclerosis patients. New Microbiol. 1997 Apr;20(2):105-14. PMID: 9208420 | Unfocused |
| 98 | Brownstein DG, Johnson EA, Smith AL. Spontaneous Reye's-like syndrome in BALB/cByJ mice. Lab Invest. 1984 Oct;51(4):386-95. PMID: 6482384 | Unfocused |
| 99 | Barinsky IF, Dementiev IV. Virological and cytogenetic studies on the involvement of bone marrow of mice in some hepatoencephalotropic viral infections. Acta Virol. 1968 Sep;12(5):464-7. PMID: 4386640 | Unfocused |

**Included: 20**

**Reviews: 7**

**Unfocused (studies with a different focus than behavioral alterations in dementia during COVID-19 pandemic and their treatment): 56**

**Editorials, comments, notes, letters without data: 12**

**Preprint:1**

**In vitro: 1**

**Non-English (papers written in languages other than English): 1**

**Rationale:1**
